# Limited cross-border infections in patients newly diagnosed with HIV in Europe

**DOI:** 10.1186/1742-4690-10-36

**Published:** 2013-04-03

**Authors:** Dineke Frentz, Annemarie M J Wensing, Jan Albert, Dimitrios Paraskevis, Ana B Abecasis, Osamah Hamouda, Louise B Jørgensen, Claudia Kücherer, Daniel Struck, Jean-Claude Schmit, Birgitta Åsjö, Claudia Balotta, Danail Beshkov, Ricardo J Camacho, Bonaventura Clotet, Suzie Coughlan, Stéphane De Wit, Algirdas Griskevicius, Zehava Grossman, Andrzej Horban, Tatjana Kolupajeva, Klaus Korn, Leondios G Kostrikis, Kirsi Liitsola, Marek Linka, Claus Nielsen, Dan Otelea, Roger Paredes, Mario Poljak, Elisabeth Puchhammer-Stöckl, Anders Sönnerborg, Danica Stanekova, Maja Stanojevic, Anne-Mieke Vandamme, Charles A B Boucher, David A M C Van de Vijver

**Affiliations:** 1 Department of Virology, Erasmus Medical Center, Rotterdam, The Netherlands; 2 Department of Medical Microbiology, University Medical Center Utrecht, Utrecht, the Netherlands; 3 Department of Microbiology, Tumor and Cell Biology, Karolinska Institutet, Stockholm, Sweden; 4 Department of Clinical Microbiology, Karolinska University Hospital, Stockholm, Sweden; 5 National Retrovirus Reference Center, Department of Hygiene Epidemiology of Medical Statistics, Medical School, University of Athens, Athens, Greece; 6 Centro de Malária e outras Doenças Tropicais, Instituto de Higiene e Medicina Tropical, Universidade Nova de Lisboa, Lisboa, Portugal; 7 Robert Koch-Institute, Berlin, Germany; 8 Statens Serum Institute, Copenhagen, Denmark; 9 Laboratory of Retrovirology, CRP-Santé, Luxembourg, Luxembourg; 10 Centre Hospitalier de Luxembourg, Luxembourg, Luxembourg; 11 Section for Microbiology and Immunology,The Gade Institute, University of Bergen, Bergen, Norway; 12 University of Milan, Milan, Italy; 13 Department of Virology, National Center of Infectious and Parasitic Diseases, Sofia, Bulgaria; 14 Hospital Egas Moniz, Centro Hospitalar de Lisboa Ocidental, Lisboa, Portugal; 15 irsiCaixa AIDS Research Institute & Lluita contra la SIDA Foundation, Hospital Universitari ”Germans Trias i Pujol”, Badalona, Spain; 16 University College Dublin, Dublin, Ireland; 17 Department of Infectious Diseases, St Pierre University Hospital, Brussels, Belgium; 18 National Public Health Surveillance Laboratory, Vilnius, Lithuania; 19 School of Public Health, Tel-Aviv University, Tel Aviv, Israel; 20 Warsaw Medical University and Hospital of Infectious Diseases, Warsaw, Poland; 21 Infectology Center of Latvia, Riga, Latvia; 22 University of Erlangen-Nuremberg, Erlangen, Germany; 23 University of Cyprus, Nicosia, Cyprus; 24 National Institute for Health and Welfare, Helsinki, Finland; 25 National Institute of Public Health, Prague, Czech Republic; 26 Molecular Diagnostics, ”Prof. Dr. Matei Bals“ Institute for Infectious Diseases, Bucharest, Romania; 27 University of Ljubljana, Ljubljana, Slovenia; 28 Medical University Vienna, Vienna, Austria; 29 Divisions of Infectious Diseases and Clinical Virology, Karolinska Institute, Stockholm, Sweden; 30 Slovak Medical University, Bratislava, Slovakia; 31 School of Medicine, University of Belgrade, Belgrade, Serbia; 32 Rega Institute for Medical Research, KU Leuven, Leuven, Belgium

**Keywords:** Europe, HIV-1, Transmission, Clusters

## Abstract

**Background:**

International travel plays a role in the spread of HIV-1 across Europe. It is, however, not known whether international travel is more important for spread of the epidemic as compared to endogenous infections within single countries. In this study, phylogenetic associations among HIV of newly diagnosed patients were determined across Europe.

**Results:**

Data came from the SPREAD programme which collects samples of newly diagnosed patients that are representative for national HIV epidemics. 4260 pol sequences from 25 European countries and Israel collected in 2002–2007 were included.

We identified 457 clusters including 1330 persons (31.2% of all patients). The cluster size ranged between 2 and 28. A number of 987 patients (74.2%) were part of a cluster that consisted only of patients originating from the same country. In addition, 135 patients (10.2%) were in a cluster including only individuals from neighboring countries. Finally, 208 patients (15.6%) clustered with individuals from countries without a common border. Clustering with patients from the same country was less prevalent in patients being infected with B subtype (*P*-value <0.0001), in men who have sex with men (*P*-value <0.0001), and in recently infected patients (*P*-value =0.045).

**Conclusions:**

Our findings indicate that the transmission of HIV-1 in Europe is predominantly occurring between patients from the same country. This could have implications for HIV-1 transmission prevention programmes. Because infections through travelling between countries is not frequently observed it is important to have good surveillance of the national HIV-1 epidemics.

## Background

Travel and migration have contributed to the world-wide spread of HIV-1. For instance, HIV was introduced in the America’s through travel and migration from Africa and Haiti in the 1960s [1]. Travel has also played a role in the early spread of HIV in East-Africa. A phylogenetic study that included geographic information found that the HIV epidemic spread more rapidly in areas in East-Africa with a good infrastructure that facilitates traveling [2]. Moreover, we recently showed that within Europe Mediterranean countries are a source of HIV-1 subtype B infections for other European countries [3].

Although travel and migration played a key role in the early spread of HIV, it is not known to what extent travel explains current transmission of HIV. On the one hand, the importance of travel may have declined over the years, because travel from sub-Saharan Africa may have decreased due to stricter European immigration laws. But also among native-born Europeans, travel may have become less important for the spread of HIV. In Europe, the HIV prevalence is generally low, and stable at 0.2% over the last decade [4] and is concentrated mainly in specific risk groups (men who have sex with men (MSM) and injection drug users) [5]. Because the HIV epidemic is well-spread in all European countries, many transmissions could take place within a country. On the other hand, the role of travel in transmission of HIV-1 may also have increased further in recent years. International travelling has become easier within Europe in the last decade because of low cost airlines and the absence of border control between most countries.

In this study we used data from the pan-European SPREAD project. SPREAD includes individuals newly diagnosed with a HIV-1 infection that are representative for the risk group and geographical distribution of the HIV epidemic in participating countries [6,7]. By performing phylogenetic analyses on this data we estimated the proportion of individuals newly diagnosed with HIV that was infected within their own country.

## Results

### Characteristics

A total of 4,260 patients newly diagnosed with HIV-1 were included. The characteristics of these patients are summarized in Table 1. The most commonly reported transmission risk groups were MSM (48%), followed by heterosexuals (35%) and injection drug users (8%). Most patients were male (79%). The most frequently found subtypes were B (66%), A (11%) and C (7%). Other subtypes or circulating recombinant forms were CRF02_AG (5%), G (3%), F (2%), and other (4%). A proportion of 1.9% of the sequences could not be classified. The proportion of subtype B was ranging among the countries between 14.3% in Latvia and 95% in Slovenia. We previously published a more detailed analysis of the subtype distribution per country [8]. Nearly one third (29%) of patients were defined as recently infected (<1 year). The median CD4 cell count 354 cells/mm^3^ (IQR: 181–540), which indicates that approximately half of the included patients were diagnosed at a stage of their infection where they were eligible to receive antiretroviral treatment.

**Table 1:** Characteristics of patients

**Characteristics**	**Categories**	**Total patients**
**Patients**		4260
**Continent of Origin, no. (%)**	Western Europe	2361 (55.4)
	Eastern Europe & Central Asia	915 (21.5)
	Sub-Saharan Africa	467 (11.0)
	Other	517 (12.1)
**Baseline values**	HIV-RNA load, mean (IQR), log copies/ml	4.8 (4.3-5.3)
	CD4 cell count, median (IQR), cells/mm^3^	354 (181–540)
	Age, mean years (IQR)	36.3 (29–42)
**Gender, no. (%)**	male	3361 (78.9)
**Risk group, no. (%)**	Men having Sex with Men (MSM)	2061 (48.4)
	Heterosexual contact	1477 (34.7)
	Injection drug use	347 (8.1)
	Other	39 (0.9)
	unknown	336 (7.9)
**CDC stage, no. (%)**	A and B	3537 (83.0)
	C	516 (12.1)
**Subtype, no. (%)**	B	2820 (66.2)
	A	477 (11.2)
	C	291 (6.8)
	02_AG	197 (4.6)
	G	137 (3.2)
	F	92 (2.2)
	others	167 (3.9)
	unassigned	79 (1.9)
	non-B	1361 (31.9)
**Duration of infection, no. (%)**	<1 year	1228 (28.8)
	1-2 years	141 (3.3)
	Unknown duration	2891 (67.9)
**TDRM, no. (%)**	present	380 (8.9)

**NOTE**. Data are no. (%) of patients, unless otherwise indicated. Characteristics describe patients from whom a baseline HIV-1 genotypic analysis was available. *CDC* Centers for disease control and prevention; *IQR* Interquartile ranges; *MSM* Men who have sex with men; *TDRM* Transmitted drug resistance mutations.

The number of patients per country of residence was for Austria 138, for Belgium 340, for Bulgaria 2, for Croatia 15, for Cyprus 55, for Czech Republic 325, for Denmark 295, for Finland 95, for Germany 685, for Greece 185, for Ireland 93, for Israel 119, for Italy 197, for Latvia 72, for Lithuania 11, for Luxembourg 52, for the Netherlands 97, for Norway 118, for Poland 193, for Portugal 238, for Romania 67, for Serbia 67, for Slovakia 23, for Slovenia 84, for Spain 351, and for Sweden 343. Table 2 compares the risk group distribution per country with surveillance data of patients newly diagnosed with HIV as reported by the European Centres for Disease Prevention and Control (ECDC). Deviations of more than 20% were found in Cyprus, Poland, Germany and Serbia. It should be noted that ECDC only included the risk group distribution for less than 30% of patients from Poland and Cyprus which can explain the strong deviation found in these countries. In Germany, we over-sampled MSM (84% in our data vs. 56% in the surveillance data from ECDC). In Serbia we over-sampled patients that acquired HIV heterosexually (49% in our data versus 25% in data from the ECDC).

**Table 2:** Comparison of the proportional HIV risk group distribution in the participating countries of the SPREAD study with proportional HIV risk group distribution collected in 2007 by the European Centres for Disease Prevention and Control (ECDC)

	***Men having Sex with Men*(*MSM*)**			***Heterosexual***			***Injecting drug users***		
**Country**	**SPREAD**	**ECDC**	**Absolute difference**	**SPREAD**	**ECDC**	**Absolute difference**	**SPREAD**	**ECDC**	**Absolute difference**
Austria	39.1%	36.9%	2.2%	47.8%	41.1%	6.7%	6.5%	15.2%	8.7%
Belgium	43.5%	27.8%	15.7%	34.4%	40.5%	6.1%	0.9%	2.0%	1.1%
Croatia	60.0%	57.7%	2.3%	20.0%	28.8%	8.8%	13.3%	3.8%	9.5%
Cyprus	49.1%	13.7%	35.4%	45.5%	14.4%	31.1%	0.0%	1.4%	1.4%
Czech Republic	41.8%	63.6%	21.8%	20.0%	23.1%	3.1%	7.7%	9.9%	2.2%
Denmark	44.7%	46.4%	1.7%	45.4%	42.5%	2.9%	5.1%	6.9%	1.8%
Finland	45.3%	36.2%	9.1%	45.3%	40.4%	4.9%	8.4%	6.9%	1.5%
Germany	84.4%	56.0%	28.4%	7.7%	24.1%	16.4%	3.5%	5.5%	2.0%
Greece	67.0%	55.5%	11.5%	21.1%	25.3%	4.2%	2.7%	2.3%	0.4%
Ireland	33.3%	20.5%	12.8%	43.0%	40.7%	2.3%	18.3%	12.8%	5.5%
Israel	39.5%	21.9%	17.6%	46.2%	48.2%	2.0%	13.4%	11.0%	2.4%
Italy	37.6%	25.1%	12.5%	53.3%	49.2%	4.1%	2.5%	8.5%	6.0%
Latvia	8.3%	4.2%	4.1%	33.3%	36.0%	2.7%	38.9%	40.2%	1.3%
Luxembourg	34.6%	36.2%	1.6%	53.8%	38.3%	15.5%	3.8%	14.9%	11.1%
Netherlands	45.4%	62.5%	17.1%	45.4%	30.2%	15.2%	7.2%	1.1%	6.1%
Norway	32.2%	31.0%	1.2%	58.5%	57.3%	1.2%	8.5%	5.2%	3.3%
Poland	37.3%	4.7%	32.6%	28.5%	10.2%	18.3%	26.9%	12.9%	14.0%
Portugal	16.0%	15.0%	1.0%	62.6%	63.5%	0.9%	16.0%	19.4%	3.4%
Romania	14.9%	3.6%	11.3%	53.7%	66.1%	12.4%	3.0%	1.8%	1.2%
Serbia	32.8%	44.0%	11.2%	49.3%	25.3%	24.0%	14.9%	13.2%	1.7%
Slovakia	65.2%	64.1%	1.1%	34.8%	30.8%	4.0%	0.0%	2.6%	2.6%
Slovenia	86.9%	81.1%	5.8%	13.1%	5.4%	7.7%	0.0%	0.0%	0.0%
Spain	53.3%	44.1%	9.2%	27.4%	39.3%	11.9%	8.3%	9.9%	1.6%
Sweden	36.4%	22.0%	14.4%	51.6%	40.7%	10.9%	9.9%	11.6%	1.7%

The numbers may not add up to 100% as the HIV risk group is not always known (most notably this distribution was reported by ECDC for <30% of patients in Cyprus and Poland).

More than half of all patients (55%) originated from Western Europe, followed by patients originating from Eastern Europe and Central Asia (22%) and from Sub-Saharan Africa (11%). A total of 3322 (77%) patients, were originating from a country in Europe. A number of 3035 (70%) patients were living in their country of origin. This ranged between 40.3% to 100%. The lowest proportions of people living in their country of origen were found in Israel (40.3%; 44% from Ethiopia), Sweden (48.2%; 4.5% from Thailand and 4.2% from Ethiopia), Norway (50.0%; 9.3% from Thailand and 5.9% in Ethiopia), and 51.1% in Ireland (7.6% from both the United Kingdom and Zimbabwe).

We found numerous differences between patients infected with a subtype B virus and patients infected with a non-B subtype virus. Not surprisingly, patients infected with a subtype B virus were less often originating from Sub-Saharan countries (0.7%) as compared to 31.7% in non-B subtype strains (*P*-value <0.0001). From this it follows that individuals harboring a subtype B strain were more often originating from European countries (89.8%) compared to 50.9% of individuals infected with a non-B strains (*P*-value <0.0001). Furthermore, patients with subtype B strains were more often MSM (71.9%) and recently infected (34.9%), than patients infected with a non-B subtype virus (13.6% and 15.9%, respectively) (both *P*-values <0.0001).

### Phylogenetic analyses

**Figure 1:**
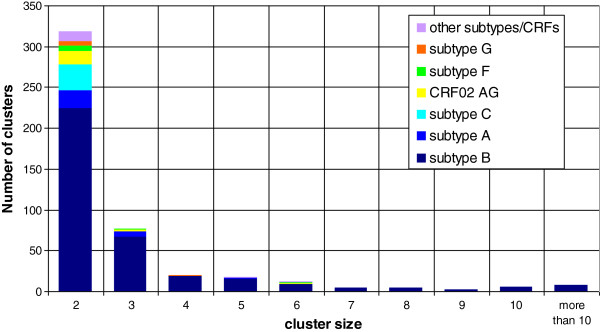
**Distribution of cluster size.** Frequency of clusters as defined in the text, of size of 2 or higher, identified by subtype.

We identified 457 clusters including 1330 persons (31.2% of all patients). The distribution of the cluster size is shown in Figure 1. The cluster size ranged between 2 and 28. Most clusters included two individuals (310 of 457 clusters, 67.8%), 112 clusters contained 3–5 persons (24.5%) and 35 clusters contained >5 persons (7.7%).

Patients that were part of a phylogenetic cluster had different characteristics as compared to patients that were not in a cluster. First, patients included in any cluster were more frequently infected through MSM (63.2% in a cluster vs. 41.3% of individuals that did not cluster, *P*-value <0.0001). Patients that were part of a cluster were more frequently infected with subtype B (82.5%; *P*-value <0.0001), recently infected (39.5%; *P*-value <0.0001) and harbouring a transmitted drug resistance mutation (10.4%, *P*-value =0.03) as compared to non-clustering patients (58.8%, 23.9%, and 8.3%, respectively). We observed a significant increase in cluster frequency among recently infected individuals from 33% in 2002 to 48% in 2007 (*P*-value = 0.002).

Of the clustering patients infected with a subtype B virus, 1013 (92.1%) patients were originating from a European country. In patients infected with a non-B subtype that were clustering, a smaller percentage of 63.5% originated from Europe (*P*-value <0.0001). Nonetheless, we found high proportions of patients originating from Europe in clustering patients infected with subtype F (25 out of 26, 96.2%), subtype A (44 out of 61, 72.1%) and subtype G (12 out of 19, 63.2%). Most of these patients infected with subtype F were living in Romania (n = 10) and Italy (n = 10) and were heterosexually infected (n = 17). Most of these patients infected with subtype A strains were living in Greece (n = 12), Latvia (n = 8), Cyprus (n = 6) and Austria (n = 6). In these patients, transmission through MSM was the most common route of transmission in patients from Greece (11 out of 12) and from Cyprus (3 out of 6), whereas in the other countries subtype A viruses were mostly transmitted among heterosexual patients. The 12 patients that were part of a cluster and were infected with subtype G were living in many different countries and were mainly heterosexual patients (n = 10).

Most patients (a number of 987, 74.2%) were part clusters that consisted only of patients originating from the same country of residence. The largest clusters were found in Poland (n = 15), Germany (n = 12 and 11), and the Czech Republic (n = 10). Among the remaining international clusters containing 343 patients, 135 (10.2%) of patients were in a cluster including only individuals from neighboring countries (the largest had 10 individuals from Denmark and Germany). Finally, 208 patients (15.6%) clustered with individuals from countries without a common border (including the largest cluster of 28 patients). The cluster size of 28 contained patients mostly living in the Czech Republic (n = 25) with two patients living in Slovakia and one patient living in Italy. Of these 28 patients, 24 patients reported to be MSM. In the 46 international clusters without a common border, most involved patients living in Spain (n = 18) or Germany (n = 15).

Table 3 shows the characteristics of the clusters and the patients involved. The proportion of patients in national clusters was different compared to international clusters for several characteristics. First, clustering with patients from the same residence country was less prevalent in patients infected with a B subtype (71.5% of all clusters) vs. non-B subtypes (87.0% of all clusters; *P*-value <0.0001). Also, MSM (68.9%) and recently infected patients (71.1%) showed less clustering with patients from the same residence country compared to heterosexuals (86.3%) or injection drug user (84.7%) (*P*-value <0.0001) and patients with a chronic or unknown duration of infection (76.2%; *P*-value =0.045). The presence or absence of transmitted drug resistance mutations did not influence the proportions of patients clustering in national clusters (74.6 and 74.2%, respectively). In a multivariate analyses, the significant difference in proportion of patients clustering in national clusters only remained for the risk group characteristic (*P*-value <0.0001).

**Table 3:** Characteristics of clusters and patients

**Characteristics**	**Category**	**All clusters, n**	**Clusters with one country of residence, n (%)**	**Clusters with neighbouring countries, n (%)**	**International clusters, n (%)**
			Characteristics of clusters		
Total		457	380 (83.2)	31 (6.8)	46 (10.1)
Subtype	Subtype B	357	291 (81.5)	26 (7.3)	40 (11.2)
	Non-B subtype	100	89 (89.0)	5 (5.0)	6 (6.0)
			Characteristics of patients in clusters		
Total		1330	987 (74.2)	135 (10.2)	208 (15.6)
Subtype	Subtype B	1100	787 (71.5)	119 (10.8)	194 (17.6)
	Non-B subtype	230	200 (87.0)	16 (7.0)	14 (6.1)
Risk group	MSM	839	578 (68.9)	103 (12.3)	158 (18.8)
	Heterosexuals	278	240 (86.3)	13 (4.7)	25 (9.0)
	IDU	85	72 (84.7)	10 (11.8)	3 (3.5)
	other	128	97 (75.8)	9 (7.0)	22 (17.2)
seroconverters	yes	523	372 (71.1)	61 (11.7)	90 (17.2)
	no	807	615 (76.2)	74 (9.2)	118 (14.6)
TDRM	yes	134	100 (74.6)	21 (15.7)	13 (9.7)
	no	1196	887 (74.2)	114 (9.5)	195 (16.3)

*MSM* Men who have sex with men; *IDU* Injection drug users; *TDRM* Transmitted drug resistance mutations.

**Figure 2:**
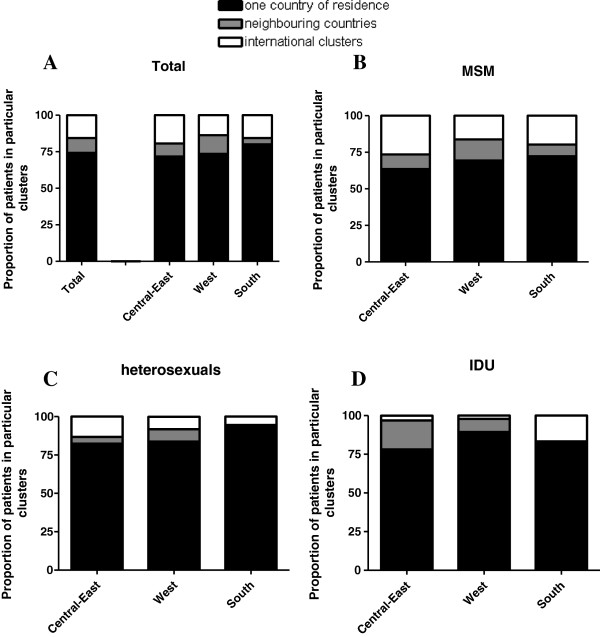
Characteristics of patients in clusters per region in all patients (A), among MSM (B), among heterosexuals (C) and among IDU (D).

In Figure 2, the proportion of patients in national clusters was observed for Central & East-, West- and South Europe, separately. We saw a statistically significant difference in proportion of patients in national clusters in Central & East- (71.7%) and West- (73.5%) and South Europe (80.0%) (*P*-value <0.001). Also, when making a distinction between the different HIV risk groups, there was a difference between Central & East-, West-, and South Europe in proportion of patients in clusters with one country of residence for MSM (*P*-value =0.007) and for heterosexuals (*P*-value =0.024), but not for IDU (*P*-value =0.20).

### Sensitivity analysis for cluster determination

We performed sensitivity analyses using different cut-off values for bootstrap values and for genetic distance (Table 4). When we changed the bootstrap value from 98% to 90%, the number of clusters found increased from 457 to 529, including 1643 persons (38.6% of all patients). The smaller bootstrap value did not change the percentage of clusters containing individuals with the same country of residence (from 83.2 to 82.0%; p = 0.67). The number of clusters which included persons from neighbouring countries was also highly comparable (7.9 and 6.8%). When we changed the genetic distance of 0.03 to a more stringent value of 0.01, the number of clusters found decreased to 327, including 811 persons (19.0% of all patients). Here, more clusters contained individuals with the same country of residence (90.8%; p = 0.002) and a 3.7% of clusters were found with neighbouring-country-patients.

**Table 4:** Sensitivity analyses on proportion of clusters containing individuals with the same country of residence

**Bootstrap value**		**Genetic distance**		
		0.01	0.02	0.03
90	Within one country	90.6	84.1	82.0
	Neighbouring country	4.5	7.7	7.9
	Without common border	4.9	8.3	10.0
98	Within one country	90.8	84.2	83.2
	Neighbouring country	3.7	6.9	6.8
	Without common border	5.5	9.0	10.1

## Discussion

In this large collection of sequences sampled from newly diagnosed individuals considering representativeness and large coverage across Europe, we found phylogenetic relationships (clusters) between isolates in one third of the study individuals. In these clusters, the vast majority of sequences were sampled from persons living in the same country. This suggests that a large part of the spread of HIV-1 in Europe can be explained by transmission of infections taking place between patients within the same country.

A strength of our study is the data collection that is performed within the SPREAD programme. The SPREAD programme is a large and sufficiently powered pan- European study that has been running since 2002. During this time the programme included patients newly diagnosed with HIV using a predefined strategy. This strategy allowed us to include patients considering representativeness for the national HIV epidemic in participating countries.

However, even though we achieved a very good overall representativeness of the European HIV-1 epidemic, we need to acknowledge that it is difficult to exclude the existence of minor sampling biases in specific countries and transmission groups. We assessed the representativeness by comparing the distribution of the transmission groups in all countries included in SPREAD with the HIV surveillance data from the European Centre for Disease prevention and Control (ECDC) (Table 2) and found that the proportional distribution of the different transmission groups was very comparable. However, compared to the data from ECDC, MSM were somewhat over-represented in some of the countries participating in SPREAD. In this study, we found MSM having a lower proportion of clustering patients from one country. An overrepresentation of MSM would therefore have resulted in a lower overall proportion of clustering patients from one country. This confirms our finding that HIV is mainly spreading within a country.

The results of this study are in agreement with phylogenetic studies performed in single European countries [9,10]. First, a phylogenetic transmission study performed in Belgium found that local onward transmission of subtype B virus contributes to an important extent to the epidemic as virtually all patients part of a transmission cluster were of Caucasian origin [9]. Second, a study from Switzerland found that clustering was segregated between different regions in the country, as transmission events occurred preferentially within the same Swiss region [10].

Our study found that patients infected with a non-B subtype virus were less often found in phylogenetic clusters (17.5%) as compared to patients infected with a subtype B virus (39.2%). This finding reflects differences between patients infected with HIV of non-B subtypes and patients infected with a B subtype. First, a much higher proportion of migrants originating from Sub-Saharan countries are infected with a non-B subtype. A Dutch modeling study showed that the migrant groups did not have a large influence on the Dutch HIV epidemic, due to the small number of migrants, their relatively moderate sexual risk behavior and low mixing with the Dutch heterosexuals [11]. This is in concordance with phylogenetic studies in Switzerland which showed that non-B subtypes are a combined result of both migration and domestic transmission [12] whereas the subtype B epidemic is mainly driven by within country transmission [10]. Second, patients infected with a non-B subtype are less frequently recently infected (<1 yr) as compared to patients infected with a subtype B virus, thus suggesting the possibility to became infected before they moved to Europe. Because non-B subtype patients are often chronically infected at time of diagnosis and have originated from many different countries, the chance of phylogenetic clustering in these patients is smaller. Also, patients infected with a non-B subtype are more often heterosexually infected. Compared to MSM, heterosexual individuals less frequently receive a HIV test. As a consequence HIV infections are less likely to be identified in a timely manner in heterosexually infected patients.

In all HIV risk groups, clustering was found mainly between patients with the same country of residence. However, differences were seen between the risk groups. MSM did less often cluster with patients from the same country than heterosexuals and injection drug users. This is also reflected in the lower percentage of seroconverters clustering within a country compared to the non-seroconverters, which could be ascribed to the fact that HIV-infected MSM are often diagnosed at an earlier stage of infection [13]. The less frequently clustering MSM suggests that MSM more often get infected during travels to other European countries whereas heterosexuals and injection drug users get infected near home. This is supported by studies reporting an association of transmission of HIV-1 in injection users with extensive local epidemics [14,15].

Sensitivity analyses showed that our findings were not distorted by the arbitrary cut-off values that were used for the bootstrap values and for the genetic distance. Using a more stringent genetic distance increased the percentage of patients clustering with patients living in the same country. Therefore, the percentage of patients clustering with patients living in the same country is at least 83.2% or higher, because the initial genetic distance used in the main analyses was taken very wide. Larger bootstrap values did not change the results in our study. Therefore, these results are generally robust and not influenced by the level of bootstrap values used in the cluster definition.

We did not have access to dense samples in which sequences from virtually all newly diagnosed HIV-infected individuals in a particular country are included. We may therefore have underestimated the size of the clusters or missed individuals for whom we currently did not identify a phylogenetically related sequence. This is the reason also why we probably estimated a large number of small clusters. Nonetheless, we still found that one out of three individuals was part of a cluster. In addition, dense sampling is expected not to have changed the results to a great extent as we achieved a very good overall representativeness of the European HIV-1 epidemic.

## Conclusions

Our findings indicate that the transmission of HIV-1 in Europe is for a large part occurring between patients from the same country. These findings have significant public health implication, as they show that a large part of all HIV-1 infections in Europe could possibly be prevented by local interventions.

## Methods

### Ethics statement

Ethical requirements are fulfilled according to the procedure described in the EC contract. The procedure differs among the 32 countries in the network according to national legislation. Briefly, for each participating hospital or collection center, approval was obtained by the institutional medical ethical review committee. Additionally, a written informed consent was obtained for each patient. In countries where a mandatory surveillance system was already established, legally no informed consent was needed. All surveillance data were made anonymous and coded at national level.

### Study population

Data came from the SPREAD programme which included newly diagnosed HIV-1 infected patients of 18 years and older who had never been exposed to antiretroviral drugs from 2002–2007. A blood sample had to be collected from each patient within six months after diagnosis. The sampling strategies were defined in close collaboration with the national public health institutes in the participating countries that had access to the latest information on national HIV epidemics. To obtain representative samples from every country, the investigators selected individuals randomly or according to the national distribution of transmission risk groups and the geographical distribution of patients with new diagnoses of HIV-1 infection. Epidemiological, clinical, and behavioral data were collected using a standardized questionnaire within six months of diagnosis. More details on the sampling strategy are provided in previous publications from the SPREAD Programme [6,7]. Within the SPREAD study, we defined patients as recently infected when patients had a duration of infection of less than one year. The duration of infection could be calculated when a last negative HIV-test was available 3 years before diagnosis. In these patients, the date of infection was estimated as the midpoint between the date of the last negative and first positive test. In addition, individuals were defined as recently infected if they had documented negative or indeterminate HIV-1 serological results up to 12 months prior to confirmation of diagnosis by western blot.

The GenBank accession numbers for the sequences used in this analysis are listed in the Appendix.

### Phylogenetics

HIV-1 subtypes were determined by the Rega subtyping tool (version 2.0) [16]. The Rega subtyping tool assesses HIV-subtypes by the construction of phylogenetic trees with group M pure reference sequences for subtypes A-D, F-H, J and K. A sequence is classified as a particular subtype when bootstrap values are >70% without recombination in the bootscan, and when they do not cluster with a circulation recombinant form with bootstrap >70%.

Isolates suggestive of intersubtype recombination in protease and reverse transcriptase fragments were analyzed by SimPlot 3.5.1 software [17]. All sequences were aligned to consensus sequences from the Los Alamos Sequence Database using Clustal W as implemented in the BioEdit software [18]. Sequences were then trimmed to equal length and the gaps were removed. In order to remove the influence of convergent evolution at antiretroviral drug resistance mutations on the phylogenetic analysis, we excluded all sites associated with major resistance according to the International AIDS Society-USA [19]. In protease these positions are 30, 32, 33, 46, 47, 48, 50, 54, 58, 74, 76, 82, 84, 88, and 90. In reverse transcriptase the following positions were excluded: 41, 62, 65, 67, 69, 70, 74, 75, 77, 100, 101, 103, 106, 108, 115, 116, 151, 181, 184, 188, 190, 210, 215, 219 and 225. This resulted in 920 nucleotides that were used for phylogenetic analysis.

Phylogenetic analyses are computationally intensive. We therefore created two different datasets in order to analyse subtype B sequences (which is the most common subtype in Europe [6,7]) separately from non-B subtype sequences. Subtype C was chosen as out-group for analysis of sequences of subtype B. Similarly, subtype B was taken as an out-group for the analysis of non-B subtypes. Phylogenetic trees were constructed using the MEGA5 integrated analysis software [20] by maximum likelihood methods under the general time-reversible model. The reliability of the maximum likelihood tree was determined using bootstrapping with 1000 replicates. To identify transmission clusters, the novel methodology for large-scale phylogeny partition was used [21]. This method identifies transmission chains by conjugating the evaluation of node reliability, tree topology and patristic distance analysis and was validated in a large Italian cohort [21].

Clustering was based on high bootstrap values (>98%) and intra-cluster average branch lengths less than 0.03 nucleotide substitutions per site [22]. We feel that these criteria are suitable for our epidemiological questions, but we acknowledge that there is no consensus on the definition of clusters. For this reason and because the cut-offs for bootstrap values and genetic distances could impact on clustering, we performed a sensitivity analysis in which clusters were defined using a less strict bootstrap value of 90%. In addition, we also did a sensitivity analysis using stricter cut-off values for the genetic distances of 0.02 and 0.01.

To study the demographics of the transmission clusters, we divided the clusters into clusters containing patients from the same country of residence, clusters with patients from countries of residence with a common border, and clusters with patients from different countries of residence which do not share a common border. We also divided Europe into three region: Central & East (Bulgaria, Croatia, Czech Republic, Latvia, Lithuania, Poland, Romania, Serbia, Slovakia, and Slovenia), West (Austria, Belgium, Denmark, Finland, Germany, Ireland, Luxembourg, the Netherlands, Norway, and Sweden), and South (Cyprus, Greece, Italy, Israel, Portugal, and Spain) to study the demographics of the transmission clusters geographically.

### Statistical analyses

Categorical data were compared using the chi-square test. Multivariate analyses as well as the time trend analysis were performed with logistic regression. The univariate analyses where included in the multivariate analyses by the forward stepwise method in the SPSS programme.

## Appendix

GenBank Accession Numbers:

AJ971093, AJ971102, AJ971103, AJ971106, AJ971107, AJ971109, AJ971114, AJ971117, AJ971122, AJ971140, AJ971143, AJ971144, AY694290, AY694313, AY694317, AY694318, AY694321, AY694322, AY694324, AY694328-AY694330, AY694338, AY694339, AY694343-AY694345, AY694350, AY694353, AY694361, AY694362, AY694377, AY694382, AY938439, AY938441-AY938447, AY938453, AY938455, AY938460, AY938463-AY938465, AY938475, AY938476, AY938482, AY938487, AY938488, AY938490, AY938492, AY938510, AY938512, AY938513, AY938517, AY938521, AY938523, AY938531, DQ974841, DQ974844, DQ974845, DQ974847, DQ974848, DQ974850, DQ974853, DQ974854, DQ974857, DQ974858, DQ974863-DQ974865, DQ974867-DQ974873, DQ974875-DQ974877, DQ974880-DQ974882, DQ974887, DQ974890, DQ974892, DQ974893, DQ974895-DQ974897, DQ974899, DQ974902-DQ974906, DQ974908, DQ974910-DQ974912, DQ974922-DQ974924, DQ974927-DQ974929, DQ974931, DQ974932, DQ974941, DQ974944, DQ974945-DQ974947, DQ974951-DQ974953, DQ974955-DQ974963, DQ974965, DQ974966, DQ974968, DQ974982-DQ974991, DQ974996-DQ974998, DQ975003, DQ975011-DQ975015, DQ975018-DQ975021, DQ975024, DQ975032, DQ975034-DQ975036, DQ975044, DQ975136, DQ975139-DQ975147, DQ975156-DQ975159, DQ975161-DQ975163, DQ975165, DQ975169, DQ975172, DQ975173, DQ975187, EU248291-EU248297, EU248299, EU248300, EU248302, EU248303, EU248305-EU248307, EU248309, EU248310, EU248312, EU248314, EU248315, EU248317, EU248320-EU248323, EU248325, EU248327, EU248329, EU248331-EU248337, EU248340, EU248341, EU248343-EU248345, EU248347-EU248360, EU248363-EU248365, EU248368, EU248371-EU248373, EU248376-EU248378, EU248382, EU248383, EU248385-EU248387, EU248389, EU248392, EU248393, EU248396, EU248399-EU248401, EU248403, EU248404, EU248406-EU248408, EU248410-EU248412, EU248415-EU248419, EU248421-EU248426, EU248428, EU248431, EU248432, EU248435, EU248439-EU248444, EU248446, EU248448, EU248449, EU248451, EU248453, EU248455-EU248457, EU248459-EU248461, EU248463-EU248466, EU248468-EU248474, EU248476, EU248477, EU248479, EU248480, EU248483, EU248485, EU248487-EU248490, EU248492, EU248494-EU248498, EU248500-EU248505, EU248507, EU248509, EU248512, EU248515, EU248517-EU248521, EU248523, EU248526-EU248569, EU248571-EU248582, EU248584-EU248588, EU673374-EU673397, FJ030767, FJ030769, FJ030771, FJ030772, FJ185113-FJ185120, FJ185122, FJ185124, FJ185125, FJ185127, GQ398826-GQ399141, GQ399143-GQ399892, GQ399894-GQ400280, GQ400282-GQ400615, GQ400617-GQ400625, GQ400627-GQ400682, GQ400684-GQ400905, GQ400907-GQ400913, GQ400915-GQ401008, GQ401010-GQ401023, JX299533-JX299579, JX299581-JX299666, JX299668-JX299780, JX299782-JX301162.

## Competing interests

The authors have no conflicts of interest to disclose.

## Authors’ contributions

DF, AMJW, CABB and DvdV designed and implemented the analysis. DF and DvdV performed the analyses. DF and DvdV drafted the manuscript. AMJW, JA, DP, ABA, OH, LBJ, CK, DS, JCS, BA, CB, DB, RJC, BC, SC, SdW, AG, ZG, AH, TK, KK, LGK, KL, ML, CN, DO, RP, MP, EPS, AS, DS, MS, AMV and CABB contributed clinical and virological data. All co-authors contributed to the interpretation of the results. All authors have read and approved the final manuscript.
